# Hospital birth volume and rurality: Associations with pregnancy outcomes among individuals with chronic hypertension

**DOI:** 10.1002/pmf2.70115

**Published:** 2025-09-23

**Authors:** Stephanie A. Leonard, Elliott K. Main, Brielle L. Formanowski, Scott A. Lorch, Ciaran S. Phibb, Sara C. Handley, Molly Passarella, Brian T. Bateman, Katy Backes Kozhimannil

**Affiliations:** ^1^ Department of Obstetrics and Gynecology Stanford University Stanford California USA; ^2^ Department of Anesthesiology Perioperative and Pain Medicine Stanford University Stanford California USA; ^3^ Division of Neonatology Children's Hospital of Philadelphia Philadelphia Pennsylvania USA; ^4^ Department of Pediatrics Perelman School of Medicine at the University of Pennsylvania Philadelphia Pennsylvania USA; ^5^ Leonard Davis Institute of Health Economics The University of Pennsylvania Philadelphia Pennsylvania USA; ^6^ Department of Pediatrics Stanford University Stanford California USA; ^7^ Health Economics Resource Center Veterans Affairs Palo Alto Healthcare System Menlo Park California USA; ^8^ Department of Health Policy Stanford University Stanford California USA; ^9^ Division of Health Policy and Management University of Minnesota Minneapolis Minnesota USA

**Keywords:** high‐volume hospitals, hypertension, low‐volume hospitals, pre‐eclampsia, pregnancy, rural hospitals, urban hospitals

## Abstract

**Introduction:**

Chronic hypertension in pregnancy has doubled in prevalence over the past 15 years, but little is known about pregnancy outcomes at hospitals with different characteristics. We evaluated the association between hospital birth volume and rurality with risk of adverse pregnancy outcomes among individuals with chronic hypertension.

**Methods:**

We conducted a population‐based study using linked vital statistics and birth hospitalization discharge data from Michigan, Oregon, South Carolina (2008–2020), and Pennsylvania (2008–2018). We classified hospitals based on federal rural–urban county classifications and annual birth volume. The primary outcome was a composite measure of adverse pregnancy outcomes, including superimposed preeclampsia or eclampsia, severe obstetric morbidities, and fetal/neonatal morbidities. We used multivariable modified Poisson regression models with hospital fixed effects and robust standard errors to estimate the risk ratios (RRs) with 95% confidence intervals (CIs) for the primary outcome and the component outcomes for each hospital group compared with high‐volume urban hospitals.

**Results:**

Among 106,991 births to individuals with chronic hypertension, the crude incidence of the primary adverse pregnancy outcome was highest in high‐volume urban hospitals (49.5%) and lowest in low‐volume rural hospitals (34.4%). Additionally, a higher proportion of individuals giving birth at high‐volume urban hospitals had a high (≥10) obstetric comorbidity score (45% vs. 24–27% at rural and low‐volume urban hospitals). After robust adjustment for clinical characteristics in regression models, however, no differences between hospital groups were evident. Among primary outcome components, only the risk of superimposed preeclampsia or eclampsia was higher in low‐volume urban hospitals (adjusted RR: 1.21; 95% CI: 1.09–1.34) and medium‐volume rural hospitals (adjusted RR: 1.26; 95% CI: 1.05–1.50).

**Conclusions:**

Adverse pregnancy outcomes among individuals with chronic hypertension were largely similar across hospital volume and rurality groups, after accounting for differences in case mix. However, superimposed preeclampsia or eclampsia was highest at medium‐volume rural and low‐volume urban hospitals, suggesting potential opportunities for improved prenatal clinical management of chronic hypertension.

## 1 INTRODUCTION

Chronic hypertension has been steadily growing in prevalence over recent decades, currently affecting approximately 4% of births in the United States [1, 2]. This condition is also implicated in a growing proportion of maternal morbidity and mortality, and disproportionately affects Black individuals—at two to three times the prevalence of other racial and ethnic groups [2, 3, 4, 5]. Chronic hypertension puts an individual at higher risk of developing preeclampsia with severe features and related complications, including fetal growth restriction and its sequelae [3, 4]. Because of these risks, the Levels of Maternal Care hospital classification system, which was developed by the American College of Obstetricians and Gynecologists (ACOG) and the Society for Maternal‐Fetal Medicine to advance risk‐appropriate care, states that individuals with “poorly controlled or complicated chronic hypertension” should give birth at a Level II–IV hospital [6]. An analysis of 2014 nationwide hospital data, however, found that 14% of individuals with preterm chronic hypertension gave birth at hospitals with an insufficient level of care [7]. This is compounded by the fact that the number of hospitals offering obstetric healthcare has been declining since at least 2004, with rural areas experiencing the largest decrease [8]. As of 2022, 52% of rural hospitals and 36% of urban hospitals do not offer obstetric healthcare [8]. It is therefore critical to understand how hospital characteristics, including obstetric volume and rurality, affect obstetric and neonatal outcomes—especially for individuals with higher risk conditions—and to identify opportunities for improvement.

Hospital obstetric volume and rurality have been associated with variation in outcomes at birth [9, 10, 11, 12]. The evidence, however, is mixed and has differed for low‐risk and high‐risk patient populations [9, 10, 11, 12, 13, 14, 15, 16], and few prior studies have considered both obstetric volume and rurality [9, 10, 11, 12]. In addition to hospital care, prenatal care is crucial for the management of chronic hypertension—and other clinical challenges—during pregnancy, and access to prenatal care may vary by rural/urban location and by hospital birth volume. ACOG has long recognized barriers to prenatal care specific to rural residents, including transportation, workforce shortages, and distance to care, which can also be barriers for higher risk patients to access subspecialty care at higher volume centers [17].

In this study, we sought to use a population‐based, multistate database to evaluate the hypothesis that lower birth volume and rurality are associated with higher rates of adverse pregnancy outcomes among individuals with chronic hypertension. The motivation of the study is to identify potential opportunities to improve clinical care and outcomes for pregnant individuals and infants affected by chronic hypertension during pregnancy.

## 2 MATERIALS AND METHODS

### 2.1 Study population

**FIGURE 1:**
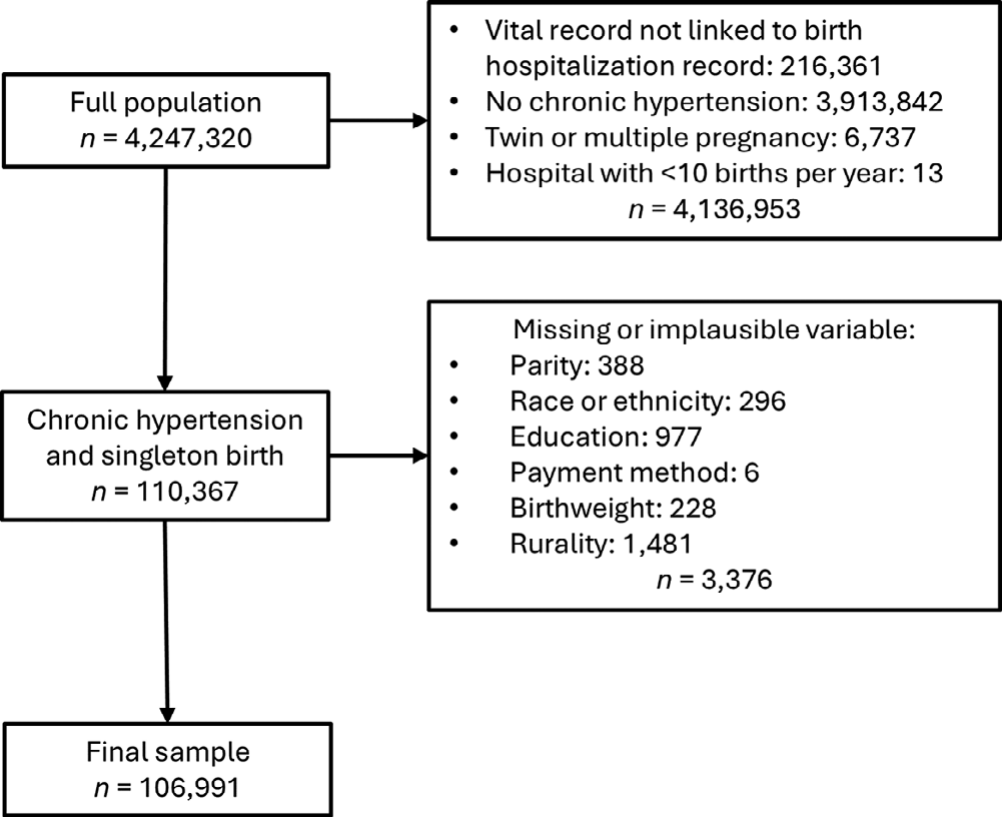
Selection of study sample.

We conducted a retrospective cohort study using linked vital statistics and birth hospitalization discharge data from Michigan, Oregon, Pennsylvania, and South Carolina. Institutional Review Board approval was obtained before the start of the study and the requirement of informed consent was waived. Births occurred between 2008 and 2020 in Michigan, Oregon, and South Carolina, and between 2008 and 2018 in Pennsylvania (most recent available year of data). Vital statistics included data collected on the US Revised Standard Certificates of Live Birth and Fetal Death, which were used by all the study states as of 2008. Previously described probabilistic and deterministic linkage techniques were used to link vital statistics and hospitalization discharge data, with a successful linkage rate of 95% [18]. We excluded unlinked records, individuals without chronic hypertension during pregnancy, twin or other multiple births, and births occurring at hospitals with fewer than 10 births/year (Figure 1). We further restricted the population to individuals with complete, plausible information on study variables of interest: maternal age, gestational age at birth, parity, race, ethnicity, educational attainment, expected method of payment for the birth hospitalization, and rurality of the birth hospital.

### 2.2 Hospital rurality and volume

The exposure of interest was hospital rurality and birth volume. We dichotomized hospitals as rural or urban based on Rural–Urban Continuum Code county classifications [19]. We categorized hospital volume based on rural or urban location following prior research that measured the distribution of birth volume in rural and urban hospitals [8, 20]. For rural hospitals, the categories included medium volume (>500 births/year) and low volume (10–500 births/year). For urban hospitals, the categories included high volume (>2000 births/year), medium volume (1001–2000 births/year), and low volume (10–1000 births/year). We used different volume thresholds for rural and urban hospitals because the volume distributions differ dramatically [21].

### 2.3 Adverse pregnancy outcomes

The primary outcome was a composite of adverse pregnancy outcomes, selected based on established, physiological links to chronic hypertension in pregnancy [3, 4, 22, 23]. The secondary outcomes in the primary composite measure were specified a priori and included superimposed preeclampsia or eclampsia, severe obstetric morbidities (acute heart failure, acute renal failure, cerebrovascular event, placental abruption, pulmonary edema, severe postpartum hemorrhage), and fetal/neonatal morbidities (stillbirth, low birth weight, preterm birth, small‐for‐gestational age). The data source and International Classification of Diseases, Clinical Modification (ICD‐CM) diagnosis codes used for each study variable are shown in Table S1. We defined severe postpartum hemorrhage as diagnosis of postpartum hemorrhage plus blood products transfusion, hysterectomy, and/or uterine repair [24]. We defined low birthweight as less than 2500 g, preterm birth as birth before 37 weeks’ gestation, and small‐for‐gestational as less than the 10th percentile for gestational age and sex using US reference charts [25].

### 2.4 Covariates

We selected confounding variables based on a directed acyclic graph (Figure S1), prior knowledge, and available data [3, 4, 5, 22, 23]. These variables included birth year, state of residence, age, method of payment for the birth hospitalization, educational attainment, parity, racial and ethnic group, and a modification of the expanded obstetric comorbidity index (removing hypertensive disorders) [26, 27]. The comorbidity index creates a weighted score from 27 comorbidities and has been validated in national data and among sociodemographic subpopulations for the outcome of severe maternal morbidity. We used the version developed for severe maternal morbidity without blood transfusion alone as an indicator. We used hospital fixed effects in the analysis to control for unobserved, time‐invariant hospital confounders [28].

### 2.5 Statistical analysis

We descriptively compared characteristics of the study population across the hospital volume and rurality groups, and calculated the incidence of each outcome in the groups. We fit multivariable modified Poisson regression models with robust standard errors to estimate risk ratios (RRs) with 95% confidence intervals (CIs) of the outcomes for each hospital group in comparison with high‐volume urban hospitals (the largest group). In the first set of models, the dependent variables included the hospital group only. In the second set of models, we added hospital fixed effects as a set of indicator variables. In the third and final set of models, we added the pre‐specified covariates: birth year, state, age, payment method, education, parity, racial and ethnic group, and the expanded obstetric comorbidity score. As a post hoc analysis to aid in the interpretation of the results, we calculated the proportion of individuals who were transferred for birth admission by hospital group. We used Stata version 18 to conduct all analyses.

## 3 RESULTS

### 3.1 Characteristics of the study sample

The study included 106,991 individuals with chronic hypertension and a singleton pregnancy (Figure 1). Among these individuals, 64,189 (60%) gave birth at 50 high‐volume urban hospitals, 23,545 (22%) at 57 medium‐volume urban hospitals, 11,288 (11%) at 96 low‐volume urban hospitals, 4283 (4%) at 24 medium‐volume rural hospitals, and 3686 (3%) at 77 low‐volume rural hospitals. One‐third of individuals were nulliparous and 73% had a condition comorbid with chronic hypertension (Table 1). Individuals giving birth at the rural hospitals tended to be younger and a higher proportion were American Indian or Alaska Native (AI/AN) or White compared with individuals giving birth at urban hospitals. The percentage of individuals with high comorbidity scores (≥10) was lower at low‐ and medium‐volume rural hospitals (24.0% and 27.1%, respectively) and low‐volume urban hospitals (26.5%) than at medium‐ and high‐volume urban hospitals (35.3% and 44.8%, respectively).

**TABLE 1:** Characteristics of study sample by hospital birth volume and rurality group.

	Total	High‐volume urban >2000 births/yr	Medium‐volume urban 1001‐2000 births/yr	Low‐volume urban 10‐1000 births/yr	Medium‐volume rural >500 births/yr	Low‐volume rural 10–500 births/yr
	*N* = 106,991	*N* = 64,189	*N* = 23,545	*N* = 11,288	*N* = 4238	*N* = 3686
**State**						
Michigan	36,823 (34.4)	23,455 (36.5)	7215 (30.6)	3639 (32.2)	1150 (26.9)	1364 (37.0)
Oregon	11,855 (11.1)	5842 (9.1)	2779 (11.8)	1836 (16.3)	674 (15.7)	724 (19.6)
Pennsylvania	32,749 (30.6)	21,010 (32.7)	7211 (30.6)	2933 (26.0)	944 (22.0)	651 (17.7)
South Carolina	25,564 (23.9)	13,882 (21.6)	6340 (26.9)	2880 (25.5)	1515 (35.4)	947 (25.7)
**Maternal Age**						
<25 years	15,097 (14.1)	8674 (13.5)	3257 (13.8)	1776 (15.7)	698 (16.3)	692 (18.8)
25–34 years	60,113 (56.2)	35,684 (55.6)	13,381 (56.8)	6422 (56.9)	2510 (58.6)	2116 (57.4)
≥35 years	31,781 (29.7)	19,831 (30.9)	6907 (29.3)	3090 (27.4)	1075 (25.1)	878 (23.8)
**Racial and Ethnic Group**						
American Indian or Alaska Native	363 (0.3)	145 (0.2)	62 (0.3)	35 (0.3)	53 (1.2)	68 (1.8)
Asian	1730 (1.6)	1210 (1.9)	350 (1.5)	132 (1.2)	25 (0.6)	13 (0.4)
Black	36,792 (34.4)	24,498 (38.2)	8042 (34.2)	2666 (23.6)	987 (23.0)	599 (16.3)
Hispanic/Latino	6614 (6.2)	4474 (7.0)	1184 (5.0)	595 (5.3)	160 (3.7)	201 (5.5)
White	58,279 (54.5)	31,793 (49.5)	13,261 (56.3)	7534 (66.7)	2971 (69.4)	2720 (73.8)
Another racial or ethnic group specified	3213 (3.0)	2069 (3.2)	646 (2.7)	326 (2.9)	87 (2.0)	85 (2.3)
**Educational Attainment**						
Less than high school degree	12,289 (11.5)	7781 (12.12%)	2364 (10.0)	1144 (10.1)	479 (11.2)	521 (14.1)
High school degree or equivalent	29,168 (27.3)	17,093 (26.63%)	6343 (26.9)	3240 (28.7)	1220 (28.5)	1272 (34.5)
Some college	38,722 (36.2)	22,430 (34.94%)	8882 (37.7)	4352 (38.6)	1699 (39.7)	1359 (36.9)
Bachelor's degree or higher	26,812 (25.1)	16,885 (26.31%)	5956 (25.3)	2552 (22.6)	885 (20.7)	534 (14.5)
**Method of Payment for Birth**						
Private insurance	54,444 (50.9)	32,099 (50.0)	12,441 (52.8)	6000 (53.2)	2252 (52.6)	1652 (44.8)
Public insurance	51,134 (47.8)	31,330 (48.8)	10,750 (45.7)	5115 (45.3)	1973 (46.1)	1966 (53.3)
Self‐pay or other	1413 (1.3)	760 (1.2)	354 (1.5)	173 (1.5)	58 (1.4)	68 (1.8)
**Nulliparous**	36,031 (33.7)	21,685 (33.8)	7947 (33.8)	3831 (33.9)	1399 (32.7)	1169 (31.7)
**Comorbidity score category**						724 (19.6)
0	28,388 (26.5)	14,771 (23.0)	6750 (28.7)	3868 (34.3)	1597 (37.3)	1402 (38.0)
1–4	21,674 (20.3)	11,937 (18.6)	5095 (21.6)	2787 (24.7)	979 (22.9)	876 (23.8)
5–9	14,820 (13.9)	8717 (13.6)	3396 (14.4)	1638 (14.5)	545 (12.7)	524 (14.2)
≥10	42,109 (39.4)	28,764 (44.8)	8304 (35.3)	2995 (26.5)	1162 (27.1)	884 (24.0)
**Chronic renal disease**	2192 (2.1)	1624 (2.5)	395 (1.7)	90 (0.8)	49 (1.1)	34 (0.9)
**Preexisting diabetes**	9297 (8.7)	6378 (9.9)	1712 (7.3)	643 (5.7)	317 (7.4)	247 (6.7)
**Preexisting cardiac disease**	4367 (4.1)	3137 (4.9)	799 (3.4)	264 (2.3)	97 (2.3)	70 (1.9)
**Thyroid disorder**	7585 (7.1)	4758 (7.4)	1570 (6.7)	743 (6.6)	280 (6.5)	234 (6.4)

*Note*: Cells display *n* (%). Cell size <12 not shown per data use agreement to protect confidentiality.

### 3.2 Incidence of the outcomes

**FIGURE 2:**
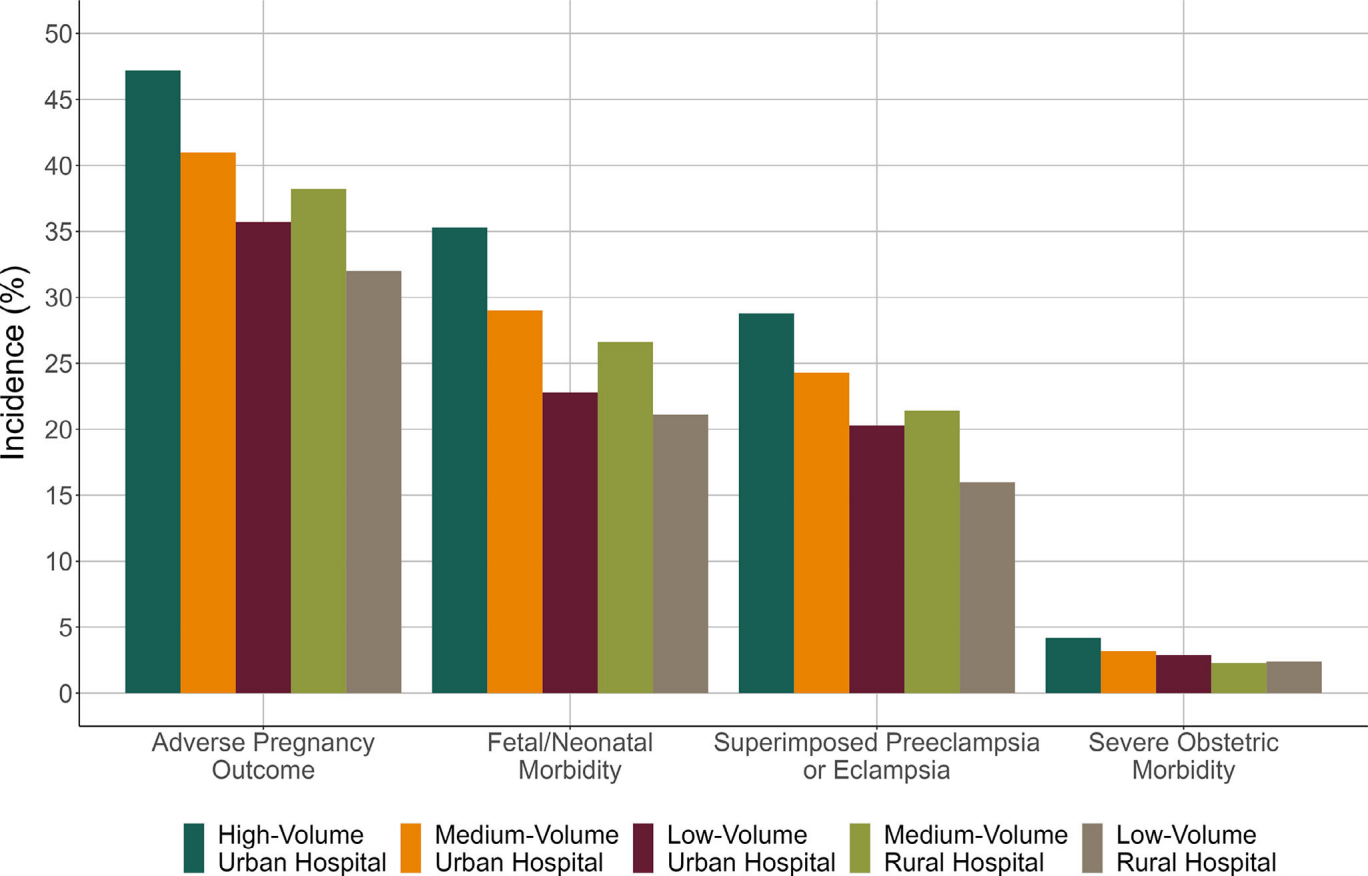
Incidence of the outcomes by hospital birth volume and rurality group among individuals with chronic hypertension.

The primary adverse pregnancy outcome occurred in 49.5% of births at high‐volume urban hospitals, 42.9% at medium‐volume urban hospitals, 37.2% at low‐volume urban hospitals, 39.9% at medium‐volume rural hospitals, and 34.4% at low‐volume rural hospitals (Figure 2). The pattern of incidence across hospital groups was the same for the component outcomes of fetal/neonatal morbidity (36.5% at high‐volume urban hospitals and 21.3% at low‐volume rural hospitals) and superimposed preeclampsia or eclampsia (31.9% at high‐volume urban hospitals and 18.9% at low‐volume rural hospitals). However, both low‐ and medium‐volume rural hospitals had the lowest incidence of severe obstetric morbidity (2.4% at both vs. 4.5% at high‐volume urban hospitals).

In the study population, incidence of the specific component outcomes was highest for superimposed preeclampsia or eclampsia (29.0%), preterm birth (23.1%), and low birthweight (18.8%) (Table 2). Superimposed preeclampsia or eclampsia, placental abruption, acute renal failure, cerebrovascular events, low birthweight, preterm birth, and small‐for‐gestational age occurred at the highest incidence at high‐volume urban hospitals and the lowest incidence at low‐volume rural hospitals. The incidence of preterm birth and low birthweight was also lower at low‐volume urban hospitals (13.0% and 10.2%, respectively) than at medium‐ or high‐volume hospitals in urban or rural areas. Differences between the hospital groups were less consistent for severe postpartum hemorrhage, pulmonary edema, and stillbirth, with the latter two outcomes occurring among fewer than 3 per 1000 births.

**TABLE 2:** Component outcomes by hospital birth volume and rurality group.

	Total	High‐volume urban >2000 births/yr	Medium‐volume urban 1001–2000 births/yr	Low‐volume urban 10–1000 births/yr	Medium‐volume rural >500 births/yr	Low‐volume rural 10–500 births/yr
	*N* = 106,991	*N* = 64,189	*N* = 23,545	*N* = 11,288	*N* = 4283	*N* = 3686
**Adverse Pregnancy Outcome (Composite of the Outcomes Below)**	49,056 (45.9)	31,783 (49.5)	10,095 (42.9)	4203 (37.2)	1709 (39.9)	1266 (34.4)
**Superimposed Preeclampsia or Eclampsia**	30,971 (29.0)	20,480 (31.9)	6276 (26.7)	2511 (22.2)	1008 (23.5)	696 (18.9)
**Severe Obstetric Morbidity Composite**	4180 (3.9)	2875 (4.5)	770 (3.3)	343 (3.0)	103 (2.4)	88 (2.4)
Placental Abruption	1858 (1.7)	1244 (1.9)	338 (1.4)	172 (1.5)	56 (1.3)	48 (1.3)
Severe Postpartum Hemorrhage	888 (0.83)	570 (0.89)	178 (0.76)	90 (0.80)	24 (0.56)	26 (0.71)
Acute Renal Failure	711 (0.66)	533 (0.83)	123 (0.52)	38 (0.34)	<12	<12
Cerebrovascular Event	493 (0.46)	360 (0.56)	97 (0.41)	21 (0.19)	<12	<12
Acute Heart Failure	457 (0.43)	342 (0.53)	69 (0.29)	32 (0.28)	<12	<12
Pulmonary Edema	132 (0.12)	87 (0.14)	28 (0.12)	15 (0.13)	<12	<12
**Fetal/Neonatal Morbidity Composite**	34,966 (32.7)	23,431 (36.5)	6980 (29.7)	2618 (23.2)	1152 (26.9)	785 (21.3)
Small for Gestational Age	14,372 (13.4)	8985 (14.0)	3043 (12.9)	1352 (12.0)	554 (12.9)	438 (11.9)
Low Birthweight	20,106 (18.8)	14,278 (22.2)	3811 (16.2)	1148 (10.2)	571 (13.3)	298 (8.1)
Preterm Birth	24,689 (23.1)	17,393 (27.1)	4707 (20.0)	1468 (13.0)	724 (16.9)	397 (10.8)
Stillbirth	221 (0.21)	124 (0.19)	48 (0.20)	34 (0.30)	<12	<12

*Note*: Cells display *n* (%). Cell sizes <12 not shown per data use agreement to protect confidentiality.

### 3.3 Multivariable regression models

In the first set of regression models (unadjusted), the estimated risk of the primary adverse pregnancy outcome, fetal/neonatal morbidity, superimposed preeclampsia or eclampsia, and severe obstetric morbidity was lower for all other hospital groups compared with the high‐volume urban hospital group (Table 3). In the second set of regression models, we controlled for time‐invariant hospital characteristics using fixed effects. In these models, the risk of the primary outcome and of fetal/neonatal morbidity did not differ between hospital groups. However, the risk of superimposed preeclampsia or eclampsia was higher for low‐volume urban hospitals (RR 1.24; 95% CI: 1.12–1.38) and medium‐volume rural hospitals (RR 1.27; 95% CI: 1.06–1.52), and results for low‐volume rural hospitals were imprecise (RR 1.15; 95% CI: 0.88–1.49). Estimates for the risk of severe obstetric morbidity were also imprecise, suggestive of lower risk at medium‐volume rural hospitals (RR 0.37; 95% CI: 0.17–0.82) and low‐volume rural hospitals (RR 0.55; 95% CI: 0.21–1.41).

**TABLE 3:** Associations between hospital birth volume and rurality group with obstetric and fetal/neonatal outcomes among individuals with chronic hypertension.

	Hospital Birth Volume and Rurality Group				
	High‐volume urban >2000 births/yr 50 hospitals *N* = 64,189	Medium‐volume urban 1001–2000 births/yr 57 hospitals *N* = 23,545	Low‐volume urban 10–1000 births/yr 96 hospitals *N* = 11,288	Medium‐volume rural >500 births/yr 24 hospitals *N* = 4283	Low‐volume rural 10–500 births/yr 77 hospitals *N* = 3686
**Adverse pregnancy outcome (Composite of the outcomes below)**					
Crude RR (95% CI)	Reference	0.87 (0.85–0.88)	0.75 (0.73–0.77)	0.81 (0.78–0.84)	0.69 (0.66–0.73)
RR (95% CI) with hospital fixed effects	Reference	0.99 (0.96–1.04)	1.07 (0.99–1.15)	1.10 (0.97–1.25)	1.04 (0.86–1.26)
RR (95% CI) with hospital fixed effects and adjusted for covariates^a^	Reference	1.00 (0.97–1.07)	1.08 (1.01–1.15)	1.09 (0.97–1.22)	0.98 (0.81–1.17)
**Fetal/neonatal morbidity**					
Crude RR (95% CI)	Reference	0.81 (0.79–0.83)	0.64 (0.61–0.66)	0.74 (0.70–0.77)	0.58 (0.55–0.62)
RR (95% CI) with hospital fixed effects	Reference	0.96 (0.91–1.01)	1.00 (0.91–1.11)	1.09 (0.92–1.29)	0.97 (0.74–1.27)
RR (95% CI) with hospital fixed effects and adjusted for covariates^a^	Reference	0.98 (0.94–1.02)	1.05 (0.96–1.14)	1.05 (0.92–1.21)	0.86 (0.67–1.11)
**Superimposed preeclampsia or eclampsia**					
Crude RR (95% CI)	Reference	0.84 (0.82–0.86)	0.70 (0.67–0.72)	0.74 (0.70–0.78)	0.59 (0.55–0.63)
RR (95% CI) with hospital fixed effects	Reference	1.07 (1.01–1.13)	1.24 (1.12–1.38)	1.27 (1.06–1.52)	1.15 (0.88–1.49)
RR (95% CI) with hospital fixed effects and adjusted for covariates^a^	Reference	1.06 (0.99–1.11)	1.21 (1.09–1.34)	1.26 (1.05–1.50)	1.06 (0.81–1.38)
**Severe obstetric morbidity**					
Crude RR (95% CI)	Reference	0.73 (0.68–0.79)	0.68 (0.61–0.76)	0.54 (0.44–0.65)	0.53 (0.43–0.66)
RR (95% CI) with hospital fixed effects	Reference	0.95 (0.79–1.13)	1.10 (0.77–1.58)	0.37 (0.17–0.82)	0.55 (0.21–1.41)
RR (95% CI) with hospital fixed effects and adjusted for covariates^a^	Reference	0.96 (0.80–1.16)	1.09 (0.77–1.56)	0.38 (0.17–0.84)	0.44 (0.17–1.17)

Abbreviations: CI, confidence interval; RR, risk ratio.

^a^ Covariates in adjusted models: birth year, state, age, payment method, education level, parity, racial and ethnic group, and obstetric comorbidity index. Hospital fixed effects were used to control for unobservable differences between hospitals.

In the third and final set of models, we adjusted for hospital fixed effects and individual‐level sociodemographic and clinical characteristics. In these models, the risk of the primary adverse pregnancy outcome was marginally higher for low‐volume urban hospitals (RR 1.08; 95% CI: 1.01–1.15) and medium‐volume rural hospitals (RR 1.09; 95% CI: 0.97–1.22) compared with high‐volume urban hospitals (Table 3). The risk of superimposed preeclampsia or eclampsia remained higher for low‐volume urban (RR 1.21; 95% CI: 1.09–1.34) and medium‐volume rural hospitals (RR 1.26; 95% CI: 1.05–1.50). The risk of fetal/neonatal morbidity remained similar across hospital groups, and results for severe obstetric morbidity were suggestive of lower risk at medium‐ and low‐volume rural hospitals. In a post hoc analysis, we found that the proportion of individuals who were transferred in for birth admission was 2.6% at high‐volume urban hospitals, 1.2% at medium‐volume urban hospitals, 0.4% at low‐volume urban hospitals, 1.1% at medium‐volume rural hospitals, and < 0.3% at low‐volume rural hospitals (number not shown because fewer than 12 individuals).

## 4 DISCUSSION

We examined pregnant individuals with chronic hypertension in a multistate dataset and, in crude results, found clear evidence of increasing incidence of adverse outcomes with increasing hospital birth volume and urban hospital location. Accounting for hospital fixed effects and individual confounders, however, revealed the highest risk among births at low‐volume urban and medium‐volume rural hospitals. This effect of adjustment on the differences between hospital groups suggests strong confounding of associations of hospital birth volume and rurality with clinical outcomes. In particular, prior studies on hospital birth volume and rurality have not employed hospital fixed effects and used different individual‐level confounders, which could contribute to the mixed findings on how these hospital characteristics may affect birth outcomes.

The majority (82%) of individuals with chronic hypertension in this study gave birth at high‐ or medium‐volume urban hospitals. These individuals were more likely than those that gave birth in other hospitals (low‐volume urban or rural hospitals) to have comorbid conditions, such as chronic renal disease or cardiac disease, and had the highest incidence of adverse outcomes. Notably, the incidence of preterm birth at low‐volume urban and low‐volume rural hospitals was less than half that at high‐volume urban hospitals. The regression results further support the conclusion that (1) the higher acuity patient population at the high‐ and medium‐volume urban hospitals drives the higher incidence of adverse outcomes and (2) referral patterns generally direct higher‐risk pregnancies to higher acuity (often higher volume) and urban hospitals. Given the range of chronic hypertension severity and the potential for worsening as pregnancy progresses, these findings illustrate the importance of continually triaging patients during pregnancy to optimize risk‐appropriate care by ensuring that the delivery site matches clinical needs.

Of interest, low‐volume urban and medium‐volume rural hospitals had the highest adjusted risk of adverse outcomes, particularly superimposed preeclampsia or eclampsia. Potential explanations are differences in the use of preventive low‐dose aspirin and antihypertensive medications, differences in written best practice guidelines, and that physicians may refer patients with hypertension that is severe or comorbid with renal or cardiac disease to high‐ and medium‐volume urban hospitals but may not refer patients with mild or moderate chronic hypertension. Risks for these individuals may still be relatively high, compared with low‐risk individuals, and resources at lower volume and rural hospitals are more limited. Structural factors, particularly distance and available transportation in rural areas, pose major barriers to referrals and transfers. Recent studies suggest that the reduction in hospitals offering obstetric healthcare has caused rural residents to travel further to give birth, with varying effects on clinical outcomes [29, 30]. A study of critical access hospitals found that many had challenges coordinating transport for obstetric emergencies, including securing a receiving facility, coordinating medical transport, and weather [31]. In this study, we were unable to identify prenatal referrals, but found that hospital transfers before birth were most common at high‐volume urban hospitals (2.6%), followed by medium‐volume urban and rural hospitals (1.2% and 1.1%, respectively), and <0.4% at low‐volume urban and rural hospitals. These results suggest that some patients with chronic hypertension initially admitted to low‐volume hospitals were later transferred to medium‐volume hospitals, in both urban and rural areas. We did not find differences between hospital birth volume and rurality groups for fetal/neonatal outcomes in multivariable analysis, which may be explained by the well‐developed regionalization of neonatal care compared to the more recent maternal levels of care.

Research and quality improvement initiatives focused on rural and low‐volume urban hospitals could potentially identify opportunities to improve healthcare quality and reduce the risk of adverse clinical outcomes among pregnant individuals with chronic hypertension. Potential strategies in need of additional research include simulation training, telemedicine consultation, and remote blood pressure monitoring [14, 32]. Adapting and implementing hypertension safety bundles for rural and low‐volume hospitals is another necessary area of research [33]. A recent qualitative study in North Carolina identified barriers and facilitators to implementation of the Severe Hypertension During Pregnancy and Postpartum Patient Safety Bundle of the Alliance for Innovation on Maternal Health at federally qualified health centers in rural areas [34]. Barriers included childcare and transportation needs of patients, and facilitators included co‐located pharmacies with needed medications and clinic champions. Adapting and evaluating innovative strategies for rural areas, such as virtual simulation training and remote blood pressure monitoring, may be beneficial, particularly paired with cost‐effectiveness analysis, given the limited financial resources of rural and lower volume hospitals [32, 35].

This study has several strengths. We used a population‐based cohort from multiple states, which supports generalizability and minimizes selection bias. We used diagnosis codes with established validity to identify health conditions and vital statistics data to identify patient characteristics, like parity, payment method, and gestational age, which are not well‐recorded in hospitalization discharge data [36, 37, 38]. Further, we used a robust analytical approach to control for measured individual‐level confounders and unmeasured hospital‐level confounders, which minimized confounding bias in the estimated associations.

Several limitations should also be considered when interpreting the study results. We used data from Michigan, Oregon, Pennsylvania, and South Carolina, which could limit generalizability of findings to other regions. Additionally, our use of hospital fixed effects does not control for time‐varying hospital characteristics, which could cause residual confounding. We also relied on diagnosis codes to identify chronic hypertension, comorbidities, and obstetric outcomes. The diagnosis codes do not differentiate severity or treatment status, and these may influence estimated associations between hospital type and outcomes. We were not able to identify individuals who were referred from smaller or rural hospitals for birth at larger volume, urban hospitals, which would further inform the interpretation of results.

## 5 CONCLUSION

In this multistate study of pregnant individuals with chronic hypertension, the risk of adverse pregnancy outcomes did not differ by hospital birth volume or rurality after adjusting for confounding variables. However, a higher adjusted risk of superimposed preeclampsia or eclampsia at medium‐volume rural and low‐volume urban hospitals indicates the importance of targeted prevention and quality improvement efforts during prenatal and intrapartum care for obstetric patients with chronic hypertension.

## CONFLICT OF INTEREST STATEMENT

Ciaran S. Phibbs and Scott A. Lorch received payment from Nelson Mullins Riley & Scarborough as expert witness, which is not related to this article. The other authors declare no conflicts of interest.

## Supporting information



Supporting Information

## Data Availability

The data that support the findings of this study are available on request from the corresponding author. The data are not publicly available due to privacy or ethical restrictions.
