# Supplementary material for: Hospital birth volume and rurality: Associations with pregnancy outcomes among individuals with chronic hypertension
Source: Pregnancy (Hoboken). 2025 Sep 23;1(5):e70115. doi: 10.1002/pmf2.70115 (PMC12857781; doi:10.1002/pmf2.70115)
Supplement: Supplementary file 1 — Supporting Information [file PMF2-1-e70115-s001.docx]

**Supplemental table 1. Details on identification of study variables.**

| **Variable** | **Data source** | **Diagnosis or procedure code(s)** | |
| --- | --- | --- | --- |
|  |  | **ICD-9-CM** | **ICD-10-CM** |
| **Chronic hypertension** | Patient discharge data | 401, 642.0, 642.7 | I10, O10.0, O11 |
| **Superimposed preeclampsia or eclampsia** | Patient discharge data | 642.4, 642.5, 642.6, 642.7 | O11, O14.0, O14.1, O14.9, O15 |
| **Severe postpartum hemorrhage^1^** | Patient discharge data |  |  |
| Postpartum hemorrhage |  | 666.0, 666.1, 666.2 | O72.0, O72.1, O72.2 |
| Blood transfusion |  | 99.0 | 30230H0, 30230K0, 30230L0, 30230M0, 30230N0, 30230P0, 30230R0, 30230T0, 30230H1, 30230K1, 30230L1, 30230M1, 30230N1, 30230P1, 30230R1, 30230T1, 30233H0, 30233K0, 30233L0, 30233M0, 30233N0, 30233P0, 30233R0, 30233T0, 30233H1, 30233K1, 30233L1, 30233M1, 30233N1, 30233P1, 30233R1, 30233T1, 30240H0, 30240K0, 30240L0, 30240M0, 30240N0, 30240P0, 30240R0, 30240T0, 30240H1, 30240K1, 30240L1, 30240M1, 30240N1, 30240P1, 30240R1, 30240T1, 30243H0, 30243K0, 30243L0, 30243M0, 30243N0, 30243P0, 30243R0, 30243T0, 30243H1, 30243K1, 30243L1, 30243M1, 30243N1, 30243P1, 30243R1, 30243T1 |
| Hysterectomy |  | 68.3, 68.4, 68.5, 68.6, 68.7, 68.39, 68.49, 68.59, 68.69, 68.79, 68.9 | 0UT90ZL, 0UT90ZZ, 0UT97ZL, 0UT97ZZ |
| Uterine repair |  | 75.5 | 0UQ9 |
| **Placental abruption** | Patient discharge data | 641.2 | O45 |
| **Cerebrovascular event** | Patient discharge data | 430, 431, 432, 433, 434, 436, 437, 671.5, 674.0, 997.02, 046.3, 348.39, 362.34, 435 | I60, I61, I62, I63, I65, I66, I67, I68, O22.52, O22.53, I97.81, I97.82, O87.3, A81.2, G45, G46, G93.49, H34.0, O22.50 |
| **Pulmonary edema** | Patient discharge data | 518.4 | J81.0 |
| **Acute renal failure** | Patient discharge data | 584.5, 584.6, 584.7, 584.8, 584.9, 669.3 | N17, O90.4 |
| **Acute heart failure** | Patient discharge data | 428.0, 428.1, 428.20, 428.21, 428.23, 428.30, 428.31, 428.33, 428.40, 428.41, 428.43, 428.9 | I50.1, I50.20, I50.21, I50.23, I50.30, I50.31, I50.33, I50.40, I50.41, I50.43, I50.810, I50.811, I50.813, I50.814, I50.82, I50.83, I50.84, I50.89, I50.9 |
| **Stillbirth** | Certificate of Fetal Death or patient discharge data | 656.40, V27.1 | O36.4, Z37.1 |
| **Preterm birth (<37 wk)** | Certificate of Live Birth or Fetal Death |  |  |
| **Small-for-gestational age (<10^th^ percentile)^2^** | Certificate of Live Birth or Fetal Death |  |  |
| **Low birthweight (<2,500 g)** | Certificate of Live Birth or Fetal Death |  |  |
| All codes subsume subsidiary codes (e.g., O10.0 includes O10.011, O10.012, O10.013...)  ^1^ Severe postpartum hemorrhage was defined as postpartum hemorrhage diagnosis plus one or more of the following indicators: blood products transfused, hysterectomy, or uterine repair, following a previously established definition. (Kramer MS, Berg C, Abenhaim H, et al. Incidence, risk factors, and temporal trends in severe postpartum hemorrhage. *Am J Obstet Gynecol.* 2013;209(5):449.e1-44.e7.)  ^2^ Talge NM, Mudd LM, Sikorskii A, Basso O. United States birth weight reference corrected for implausible gestational age estimates. Pediatrics 2014;133:844–53. | | | |

**Supplemental Figure 1. Directed acyclic graph used in selection of covariates.**

**
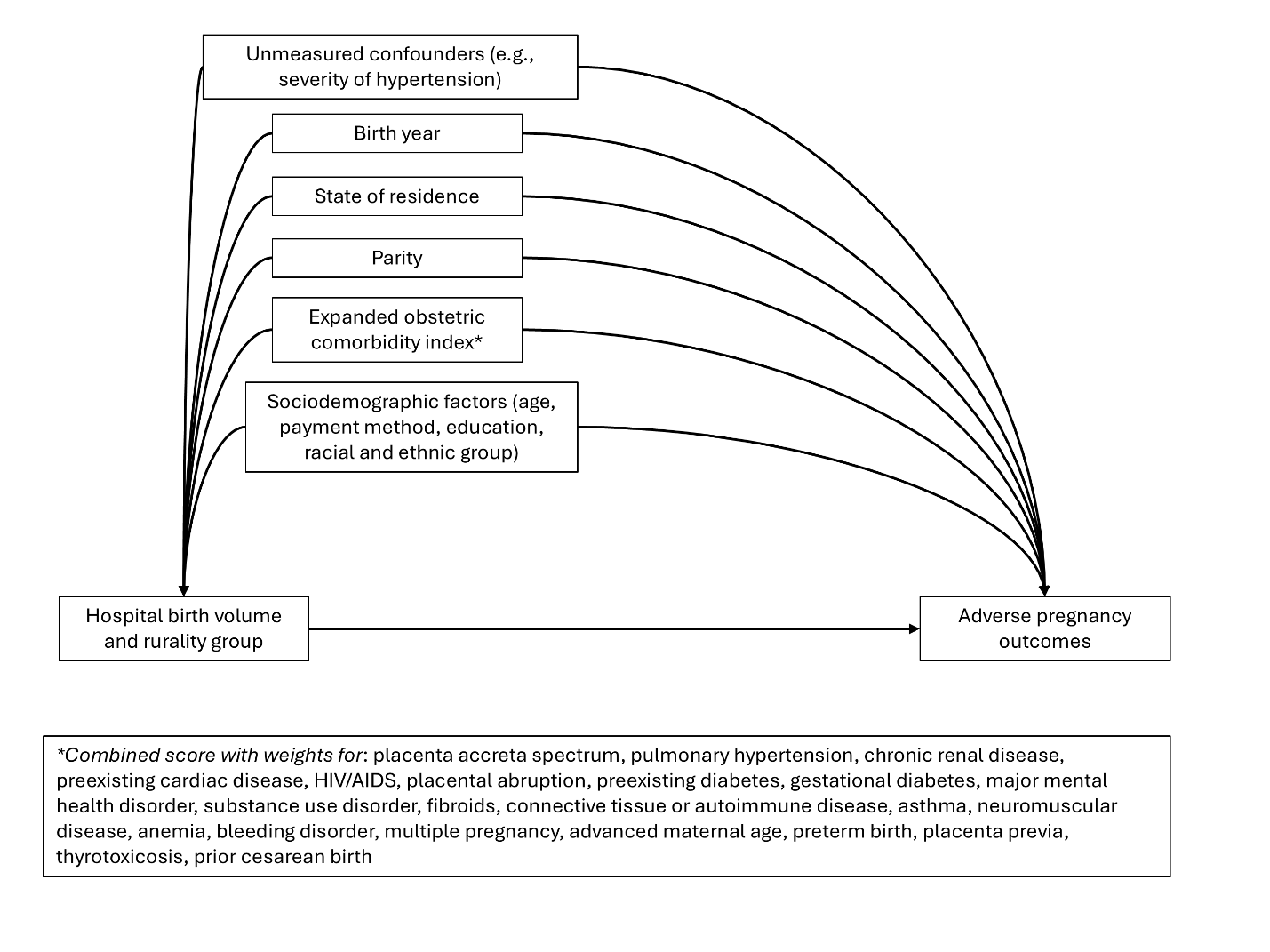
**
